# Retrospective case–case study investigation of a significant increase in *Cryptosporidium* spp. in England and Wales, August to September 2023

**DOI:** 10.2807/1560-7917.ES.2025.30.9.2400493

**Published:** 2025-03-06

**Authors:** Sarah V Williams, Eve Matthews, Thomas Inns, Christopher Roberts, Joshua Matizanadzo, Paul Cleary, Richard Elson, Chris J Williams, Reece Jarratt, Rachel M Chalmers, Roberto Vivancos

**Affiliations:** 1 UK Field Epidemiology Training Programme, United Kingdom Health Security Agency (UKHSA), London, United Kingdom; 2 Thames Valley Health Protection Team, UKHSA, Chilton, United Kingdom; 3 Field Services, UKHSA, Liverpool, United Kingdom; 4 Public Health Wales, Cardiff, United Kingdom; 5 Gastrointestinal Pathogens Unit, UKHSA, London, United Kingdom; 6 School of Environmental Sciences, University of East Anglia, Norwich, United Kingdom; 7 National Institute for Health and Care Research (NIHR), Health Protection Research Unit in Gastrointestinal Infections, Liverpool, United Kingdom; 8 NIHR Health Protection Research Unit in Emergency Preparedness and Response, London, United Kingdom; 9 Cryptosporidium Reference Unit, Public Health Wales, Swansea, United Kingdom; 10 Swansea University Medical School, Singleton Park, Swansea, United Kingdom; 11 NIHR Health Protection Research Unit in Emerging and Zoonotic Infections, Liverpool, United Kingdom; 12 Warwick Medical School, University of Warwick, Coventry, United Kingdom

**Keywords:** *Cryptosporidium*, United Kingdom, travel, case–case study, outbreaks, surveillance

## Abstract

**Background:**

Laboratory surveillance detected an unprecedented increase in *Cryptosporidium* spp. (predominantly *Cryptosporidium hominis*) in England and Wales in August 2023. Cases are not routinely followed up in all of England and Wales, and initial investigations identified no common exposures.

**Aim:**

To perform a retrospective case–case study investigation of the increase in *Cryptosporidium* spp. in England and Wales.

**Methods:**

We conducted an unmatched case–case study with 203 cases of laboratory-confirmed *C. hominis* and 614 comparator cases of laboratory-confirmed *Campylobacter* spp. reported between 14 August and 30 September 2023. We fitted a multilevel logistic regression model, with random intercepts for geographical region, to estimate adjusted odds ratios (aOR) for exposures. We present the final model as aOR and 95% confidence intervals (CI).

**Results:**

Multivariable analysis identified associations with swimming pool use (aOR: 5.3, 95% CI: 2.3–9.3), travel to Spain (aOR: 6.5, 95% CI: 3.5–12.3) and young age, with children 0–4 years having the strongest association of being a case (aOR: 3.6, 95% CI: 1.5–8.6). We also identified associations with swimming in a river, and travel to France or Türkiye, but there was low frequency of exposure among cases and comparator cases.

**Conclusions:**

Following the largest recorded increase of *Cryptosporidium* spp. and in particular *C. hominis* cases in England and Wales, we identified several exposures, suggesting that causation was likely to be multifactorial. We recommend development of a standardised questionnaire to enable rapid investigation of future case increases, which will improve existing surveillance and inform public health actions.

**Key public health message**

**What did you want to address in this study and why?**

The parasite *Cryptosporidium* causes cryptosporidiosis, a gastrointestinal infection resulting in diarrhoea usually lasting ~2 weeks. In Autumn 2023, we identified the largest increase to date of *Cryptosporidium* spp. reports in England and Wales (mostly *Cryptosporidium hominis*)*.* We wanted to determine exposures or behaviours linked with *C. hominis* infection to better understand the outbreak causes and identify actions to prevent future outbreaks.

**What have we learnt from this study?**

The increase in number of cases of *C. hominis* followed unusual weather patterns in summer 2023. We identified links with international travel and swimming pool use of the 203 cases. This suggests that there were multiple causes for the increase in numbers of cases. There was some evidence that over time an increasing number of cases were due to transmission within England and Wales rather than from infection in another country.

**What are the implications of your findings for public health?**

Further work is needed to understand the impact of other factors including COVID-19 and weather patterns on cryptosporidiosis across different countries. Existing surveillance in England and Wales should be improved with the development of a standardised questionnaire for investigation of cases, and clear documentation and training for outbreak investigation teams to enhance questionnaire completion.

## Introduction

Cryptosporidiosis is a gastrointestinal disease caused by the *Cryptosporidium* parasite. Infections result in self-limiting diarrheal disease usually lasting 2–26 days with symptoms ranging from mild to severe [1]. Some patients experience recurrent symptoms for longer periods [1] with some still experiencing gastrointestinal and joint-related symptoms several years later [2], and immunocompromised patients can develop severe and persistent illness [3]. *Cryptosporidium* is transmitted through ingestion of oocysts via the faecal–oral route. The incubation period is usually 5–7 days but very rarely can be as long as 28 days [1].

The most common species of *Cryptosporidium* infecting humans are *Cryptosporidium hominis* and *Cryptosporidium parvum* [4]. *Cryptosporidium hominis* is associated with anthropogenic transmission pathways and case numbers peak in the United Kingdom (UK) in late summer and autumn [4]. *Cryptosporidium parvum* is associated with zoonotic transmission pathways and cases peak in the UK in spring [4].

Previous outbreaks have been attributed to transmission through person-to-person routes [5], animal contact [6], contaminated food products including salad leaves [7,8] and raw and pasteurised milk [9], contaminated drinking water supplies [10] and contaminated recreational waters [11,12]. A majority of outbreaks in the UK are linked to either animal contact activities at open or commercial farms or to swimming pools; the latter are predominantly caused by *C. hominis* [4] and tend to be localised. Waterborne outbreaks are attributed to oocyst chlorine resistance and challenges with elimination through filtration [13]. Food-borne outbreaks are described less frequently, though a large and nationally distributed outbreak of *C. parvum* in the UK was associated with consumption of salad leaves [7].

In England and Wales, the routine surveillance of cryptosporidiosis relies on mandatory laboratory notification, with *Cryptosporidium* positive stool samples from local laboratories referred to the national *Cryptosporidium* Reference Unit (CRU, Swansea, UK) for genotyping, while in Europe most cases are not identified at the species level. In England, the routine collection of exposure information in cryptosporidiosis cases varies by region, while in Wales there is a standard questionnaire for gastrointestinal disease.

In Autumn 2023, routine laboratory surveillance conducted by the UK Health Security Agency (UKHSA) in England and Public Health Wales in Wales, identified an unprecedented increase in reports of *Cryptosporidium.* The exceedance system in England and Wales [14] was triggered by an increase beginning in the International Organisation for Standardisation (ISO) week 33 2023 and peaking in ISO weeks 36 and 37 2023, mainly driven by increase in cases of *C. hominis* infections [15]. Peake et al. (2023) provided early descriptive analysis of case questionnaire data but did not identify any commonalities [15]. Therefore, we carried out a case–case study aiming to identify exposures associated with an increased odds in cases of *C. hominis* compared with cases of *Campylobacter* spp.

## Methods

### Study design

We carried out a retrospective unmatched case–case design, with cases of *Campylobacter* spp. as comparator cases. 

### Definitions

A case was defined as any individual who had *Cryptosporidium* identified in a stool sample by PCR, enzyme immune assay or stained microscopy at a diagnostic laboratory, further characterised by real-time PCR of the A135 gene as *C. hominis* [16], with sample date from 14 August to 30 September 2023 inclusive, who was resident in England or Wales, with a completed surveillance questionnaire, and who had not reported contact with anyone with gastrointestinal symptoms in the 14 days before onset of their symptoms.

A comparator case was defined as any individual who had *Campylobacter* spp. identified in a stool sample by PCR or culture at a diagnostic laboratory, with a sample date from 14 August 2023 to 30 September 2023 inclusive, who was resident in England and Wales and who had not reported contact with anyone with gastrointestinal symptoms in the 14 days before onset of their symptoms.

### Sample size and sampling frame

There were 206 *C. hominis* cases in England and 80 cases in Wales from the routine surveillance systems during the study period. Based on three per case, the required comparator case sample sizes were 618 in England and 240 in Wales.

In England, comparator cases were extracted from the Second-Generation Surveillance System (SGSS), which holds all reported laboratory results in England. In Wales, comparator cases were extracted from the Welsh case and incident management system Tarian, which contained all laboratory-confirmed Welsh *Campylobacter* spp. cases who had completed a standard GI Illness Exposure Investigation Form.

Simple random sampling was used to select comparator cases from these sampling frames.

Assuming a 33% response rate to study questionnaires sent by letter, we invited 1,854 comparator cases to complete the study questionnaire in England. Welsh cases had already completed questionnaires.

### Data sources

In England, there are no standardised exposure data collection procedures for *Cryptosporidium* cases. We developed an online standardised electronic study questionnaire for English cases and comparator cases for exposures in the 14 days before illness onset (Peake et al. 2023 [15]). Data collection began in ISO week 38 (18 September 2023) for English cases, and comparator cases were invited to be participants via a letter sent by UKHSA using the GOV.UK Notify service to complete the questionnaire in ISO week 42 (16 October 2023). The survey was closed, and data extracted by the study team in ISO week 44 (30 October 2023).

Questionnaire administration varied by region in England, some using self-administration and some interviewer-led by either UKHSA Health Protection practitioners or local authority environmental health officers.

No additional data collection was required for Welsh *Campylobacter* spp. and *Cryptosporidium* spp. as they routinely complete an interviewer-led GI Illness Exposure Investigation Form.

The questionnaire for English cases and the investigation form were comparable, collecting data on demographics, foreign travel, food and water exposures, and interaction with animals. Consumption of bottled water was available for English cases only.

### Analysis

We undertook all data cleaning, manipulation and analysis using R version 4.2.2 (R Foundation). Differences in characteristics between cases and comparator cases were investigated using the Pearson’s Chi-square test or Fisher’s exact test for categorical variables, and the Student’s t-test or a non-parametric test for continuous variables. A single variable analysis was conducted for exposure variables using logistic regression to determine crude odds ratios (ORs) with 95% confidence intervals (CI). Only exposure variables with a minimum of five positive responses among cases were included in the single variable analysis and multivariable analysis. The variables for pets, animal contact, international travel and swimming or water activity are composite variables of pet ownership for different types of animals or animal contact, all international travel destinations and all different swimming and water activities, respectively. These were not included in the final model since they have substantial overlap with other variables.

For the multivariable model, we used a backwards stepwise approach to include exposure variables which had p values of 0.20 or less and ORs > 1 in the single variable analysis, and which are biologically plausible. Sex and age group were included as a priori confounders in the model, and we used the likelihood ratio test to determine if a model was a better fit than the previous step. Generalised linear mixed-effects models were estimated; geography, defined as a region within England or at country level for Wales, was included as a random effect in the model. The final model for England and Wales is presented as adjusted ORs with 95% CI.

Further information on the methods used for sub-analyses, sensitivity analyses and convergence of the models are included in the Supplementary Materials S1.

## Results

In total, 203 cases met the case definition for a *C. hominis* case and were included in the study, of which 152 were in England and 51 in Wales. For the case–case investigation, 614 comparator cases were included in the study, 461 in England and 153 in Wales, which met the a priori target ratio of three comparator cases to one case.

### Descriptive epidemiology

*Cryptosporidium hominis* cases were younger (mean age: 21 years; range: 0–76 years) than comparator cases, with a higher proportion of females in the 25–34 years and 35–44 years age groups and males in the 0–4 years and 5–15 years age groups (see Table 1). Further information on the age and sex distribution of cases is provided in the Supplementary Materials S2. Comparator cases were generally older (mean age: 51 years; range: 1–95 years) and had an even sex split in each age category. There was a percentage difference of 31% for the 45–64 years age group and 27% for the 65 years and older age group between the cases and comparator cases. Table 1 shows the characteristics of cases and comparator cases recruited to the study in further detail.

**Table 1:** Counts and proportions of characteristics of *Cryptosporidium hominis* cases and *Campylobacter* spp. comparator cases, England and Wales, 14 August–30 September 2023 (n = 817)

Characteristics	Comparator cases (n = 614)		Cases (n = 203)		p value
	n	%	n	%	
**Country **					
Wales	153	24.9	51	25.1	> 0.9^a^
England	461	75.0	152	74.9	
**ISO specimen collection week, 2023 **					
33	86	14.0	10	4.9	< 0.001^a^
34	70	11.4	21	10.3	
35	71	11.6	34	16.7	
36	82	13.4	32	15.8	
37	113	18.4	61	30.0	
38	96	15.6	36	17.7	
39	96	15.6	9	4.4	
**Sex**					
Male	317	51.6	95	46.8	0.3^b^
Female	295	48.0	108	53.2	
Other	2	0.3	0	0	
**Age (years)**					
Mean (SD)	50.8 (21.2)		21.1 (17.7)		< 0.001^c^
Median (IQR)	56.0 (37.0–66.0)		13.0 (6.0–35.0)		
Range	1.0–95.0		0.0–76.0		
**Age group (years) **					
25–34	45	7.3	28	13.8	< 0.001^a^
0–4	20	3.3	40	19.7	
5–15	36	5.9	65	32.0	
16–24	32	5.2	16	7.9	
35–44	62	10.1	33	16.3	
45–64	244	39.7	18	8.9	
≥ 65	175	28.5	3	1.5	
**Geography^d^**					
East Midlands	35	5.7	6	3.0	< 0.001^a^
East of England	56	9.1	8	3.9	
London	39	6.4	4	2.0	
North East	29	4.7	34	16.7	
North West	57	9.3	43	21.2	
South East	96	15.6	22	10.8	
South West	56	9.1	8	3.9	
Wales	153	24.9	51	25.1	
West Midlands	47	7.7	23	11.3	
Yorkshire and Humber	46	7.5	4	2.0	

IQR: interquartile range; SD: standard deviation.

^a^ Pearson's Chi-square test.

^b^ Fisher's exact test.

^c^ Wilcoxon rank sum test.

^d^ Nine regions in England, Office of National Statistics (ONS): https://www.ons.gov.uk/methodology/geography/ukgeographies/administrativegeography/england

Comparator case specimen collection dates were more evenly distributed across the study period, compared with a peak in week 37 for cases. More comparator cases were recruited in the south and East of England regions compared with cases. The North East and North West of England regions are particularly under-represented in the comparator case group.

### Analytical epidemiology

In the single variable analysis (Table 2), *C. hominis* cases had increased odds of being aged 0–4 years or aged 5–15 years compared with comparator cases, *Campylobacter* spp. The age group 25–34 years was chosen as the age reference group because of having the fewest cases among adults. Cases had increased odds of taking part in water activities including swimming in a pool, sea, river or other location, with swimming in a pool having the highest odds. There were also increased odds of travel to France, Greece, Spain and Türkiye, with travel to Spain having the highest odds. Cases of *C. hominis* had reduced odds for all the food exposures compared with comparator cases.

**Table 2:** Single variable analysis of *Cryptosporidium hominis* cases and *Campylobacter* spp. comparator cases, England and Wales, 14 August–30 September 2023 (n = 815)

Characteristics	Comparator cases (n = 612)		Cases (n = 203)		OR	95% CI	p value
	n	%	n	%			
**Sex^a^**							
Male	317	51.8	95	46.8	Reference		
Female	295	48.2	108	53.2	1.22	0.89–1.68	0.2
**Age group (years) **							
25–34	44	7.2	28	13.8	Reference		
0–4	20	3.3	40	19.7	3.14	1.55–6.53	0.002
5–15	36	5.9	65	32.0	2.84	1.53–5.35	0.001
16–24	32	5.2	16	7.9	0.79	0.36–1.68	0.5
35–44	62	10.1	33	16.3	0.84	0.44–1.58	0.6
45–64	243	39.7	18	8.9	0.12	0.06–0.23	< 0.001
≥ 65	175	28.6	3	1.5	0.03	0.01–0.08	< 0.001
**Exposure**							
Mains water supply used	381	62.3	89	43.8	0.47	0.34–0.65	< 0.001
Private water supply used	18	2.9	5	2.5	0.83	0.27–2.12	0.7
Swam (any location)	145	23.7	125	61.6	5.16	3.69–7.27	< 0.001
Other water-based activity	44	7.2	37	18.2	2.88	1.79–4.60	< 0.001
Swam in pool	57	9.3	93	45.8	8.23	5.61–12.2	< 0.001
Swam in sea	13	2.1	11	5.4	2.64	1.14–6.00	0.020
Swam in river	4	0.7	6	3.0	4.63	1.31–18.3	0.019
Day trip	124	20.3	29	14.3	0.66	0.42–1.01	0.060
Pets in household	326	53.3	110	54.2	1.04	0.75–1.43	0.8
Visited a farm	55	9.0	27	13.3	1.55	0.94–2.52	0.078
Any animal contact	256	41.8	84	41.4	0.98	0.71–1.35	> 0.9
Contact with dogs	184	30.0	67	33.0	1.15	0.81–1.61	0.4
Contact with cats	95	16.5	26	12.8	0.80	0.49–1.26	0.3
Contact with rabbits	4	0.7	5	2.5	3.84	1.01–15.6	0.047
Consumed fast food	356	58.2	98	48.3	0.67	0.49–0.92	0.014
Consumed salad leaves	302	49.3	57	28.1	0.40	0.28–0.56	< 0.001
Consumed berries	331	54.1	75	36.9	0.50	0.36–0.69	< 0.001
Consumed pre-packaged sandwiches	135	22.1	17	8.4	0.32	0.18–0.54	< 0.001
Consumed any milk or dairy products	449	73.4	83	40.9	0.25	0.18–0.35	< 0.001
Travel to United Kingdom	149	24.3	35	17.2	0.65	0.43–0.96	0.037
Travel international	134	21.9	132	65.0	6.63	4.71–9.42	< 0.001
Travel to France	7	1.1	12	5.9	5.43	2.15–14.8	< 0.001
Travel to Greece	6	1.0	5	2.5	2.55	0.73–8.56	0.13
Travel to Spain	38	6.2	69	34.0	7.78	5.05–12.2	< 0.001
Travel to Türkiye	11	1.8	17	8.4	4.99	2.32–11.2	< 0.001

CI: confidence interval; OR: odds ratio.

^a ‘^Other’ group not included.

Table 3 shows the final multivariable model, with geography included as a random effect and age group and sex included as a priori confounders. Children aged 0–4 years and children aged 5–15 years had higher odds of being a case than adults aged 25–34 years. The aOR of being a case among those who had travelled to Spain, Türkiye or France were higher than those who had not travelled, although only 17 and 12 cases had travelled to Türkiye and France, respectively, during their exposure period. Swimming in a pool was also associated with increased odds of being a case; there were 93 cases who reported this exposure, as was swimming in a river, with 6 cases having this exposure. Results of the sub-analyses and sensitivity analyses are reported in the Supplementary Materials S2.

**Table 3:** Final multivariable model for exposures associated with being a *Cryptosporidium hominis* case, England and Wales, 14 August 2023–30 September 2023 (n = 815)

Characteristics	Comparator cases (n = 612)		Cases (n = 203)		OR	95% CI	p value
	n	%	n	%			
**Sex** ^a^							
Male	317	51.8	95	46.8	Reference		
Female	295	48.2	108	53.2	2.06	1.25–3.40	0.004
**Age group (years) **							
25–34	44	7.2	28	13.8	Reference		
0–4	20	3.3	40	19.7	3.58	1.49–8.59	0.004
5–15	36	5.9	65	32.0	2.29	1.05–5.02	0.038
16–24	32	5.2	16	7.9	0.62	0.24–1.61	0.3
35–44	62	10.1	33	16.3	0.67	0.31–1.44	0.3
45–64	243	39.7	18	8.9	0.08	0.03–0.18	< 0.001
≥ 65	175	28.6	3	1.5	0.04	0.01–0.14	< 0.001
**Exposures**							
Swam in pool	57	9.3	93	45.8	5.26	2.97–9.31	< 0.001
Swam in river	4	0.7	6	3.0	7.08	1.24–40.5	0.028
Travel to France	7	1.1	12	5.9	12.3	3.21–46.8	< 0.001
Travel to Spain	38	6.2	69	34.0	6.53	3.46–12.3	< 0.001
Travel to Türkiye	11	1.8	17	8.4	4.07	1.30–12.8	0.016

CI: confidence interval; OR: odds ratio.

^a ‘^Other’ group not included.

The Figure illustrates the relationship between international travel (all destinations) with swimming pool use, by the ISO specimen collection week. While most cases had international travel and swimming pool use, the proportion of cases with no international travel increased over time.

**Figure:**
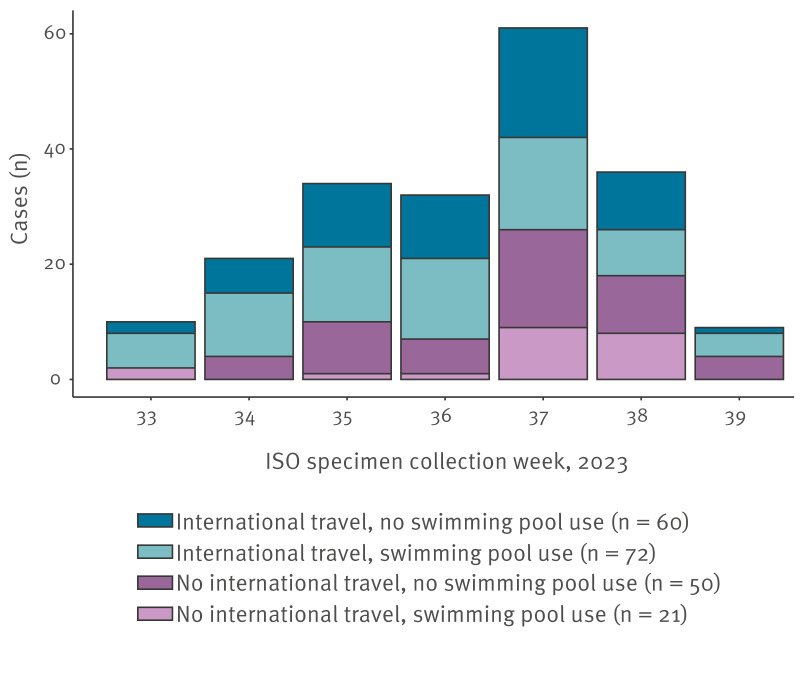
*Cryptosporidium hominis* cases with international travel or swimming pool use by ISO specimen collection week, England and Wales, 14 August–30 September 2023 (n = 203)

## Discussion

We conducted a case–case study to identify exposures associated with cases of *C. hominis* compared with *Campylobacter* spp. and identified associations with swimming pool use, swimming in a river, travel to Spain, Türkiye or France, and age. The strongest associations were with swimming in rivers and travel to France, but these exposures were infrequent for both cases and comparator cases. Over time, a greater proportion of cases had no international travel, with some having swimming pool exposure in England and Wales, which suggests that the outbreak progressed from importation of cases to localised transmission.

An increase in cases of cryptosporidiosis has also been described in other European countries [17-19]. In 2023, a study undertaken in Germany also identified a relationship with swimming pools, and a significant increase in the proportion of cases having travelled to Croatia in 2023 [18]. However, in our study in England and Wales, only one case had travelled to Croatia (data not shown), and Spain was the most reported travel destination followed by Türkiye and France. Spain also observed a marked increase in cryptosporidiosis cases in 2023 [19]. Spain is the most popular holiday destination for UK travellers, accounting for 21% of all visits abroad in 2023, followed by France [20]. Türkiye was the eighth most popular destination for UK international travel while Croatia was not a top 10 destination in 2023 [20]. It is unlikely that the travel associations that we observed are artefacts of common travel destinations within the UK population, as otherwise the exposure should be similar across cases and comparator cases. However, it is possible that travel associations reflect increased swimming pool use, which would also be supported by the findings of Schoeps et al. in Germany [18] and by Peñuelas Martinez et al. in Spain [19]. Other studies, including one in the north of England on an increase in *Cryptosporidium* infections in 2012 [21], have previously demonstrated an association with cryptosporidiosis and international travel, although the percentage of travel-related cases was similar to previous years.

The results of our study are supported by existing research on cryptosporidiosis. Most *C. hominis* outbreaks in England and Wales are related to the use of swimming pools [4], and an international review reported that recreational waters and swimming pools were the setting for 92% of waterborne cryptosporidiosis outbreaks [22]. Unlike *Campylobacter* spp., *Cryptosporidium* is resistant to chlorine [13] and so the association with swimming pools that we found is not surprising.

The European Centre for Disease Prevention and Control (ECDC) reported in October 2023 multi-country increases in cases of cryptosporidiosis across Europe [23], though most cases were not identified to the species level. The widespread distribution of cases’ travel locations, across multiple countries and a variety of locations within countries, implies widespread transmission rather than single point source outbreak. An explanation for this is not clear. There was disruption of *C. hominis* transmission during the COVID-19 pandemic [24] with very low numbers of cases affecting immunity levels. Greater numbers of people may have travelled abroad in summer 2023 after much reduced foreign travel during COVID-19, with more people being exposed to risk factors for *C. hominis* such as recreational water use. It is also possible that weather patterns in Europe throughout the summer of 2023 increased the risk of contamination of drinking water and recreational water. Previous outbreaks have been linked to contamination of the public water supply by human sewage [10], and a *C. hominis* outbreak in Germany was linked to river flooding following a hot summer [25] but the mechanism for widespread transmission in Europe summer 2023 is unclear. The weather was characterised by hot periods followed by rain [26], with June particularly hot and dry, followed by wet weather in July and August [26], the months immediately preceding and during the exceedance of *Cryptosporidium* spp. observed in the UK. Copernicus, the European Union (EU)’s climate change information service, reported high river flow leading to discharge anomalies in June–August 2023, particularly in Spain and northern and western France [26]. A study in the UK previously demonstrated that weather patterns play an important role in the seasonality of cryptosporidiosis [27] and there is evidence that an increase in temperature and precipitation are linked to increases in cryptosporidiosis [28]. If there are more unusual weather events, then we may see future seasonal exceedances of *Cryptosporidium* and other waterborne diseases and, thus, further exploration of the relationship with weather patterns would be of value.

The increase of *Cryptosporidium* spp. seen across the UK was driven by *C. hominis* as expected at the time of year [4,27,28]). International travel from the UK in 2023 had increased from 2022 but was not yet back to pre-COVID-19 pandemic levels [20] and so it is unlikely that it has fully recovered, making it unclear as to why the excessive size and unusual pattern of increase of cryptosporidiosis was seen in 2023.

There are some limitations to our study. Firstly, case–case studies are subject to recall bias. Our comparator cases were also cases of a gastrointestinal illness, and so they should have similar recall of events and exposures as the cases of *C. hominis*. However, in England, the comparator cases completed the questionnaire a few weeks after their illnesses, which may have affected their recall. In addition, some important exposures may not have been included in the questionnaire for both cases and comparator cases. Comparator cases were also generally older than cases with fewer children included, and so the behaviours and exposures of the two groups may be different.

Secondly, our choice of *Campylobacter* spp. cases as comparator cases makes it difficult to identify food associations for the cases of *C. hominis* identified in earlier work [15]. In our single variable analysis, cases had reduced odds of food exposures compared with comparator cases, which is likely due to a stronger association between *Campylobacter* spp. and food exposures than for *C. hominis*. However, other sources of comparator cases or controls, such as market research, have issues and data on *Campylobacter* spp. cases were easily accessible.

Thirdly, in our overall model, we combined cases and comparator cases from England and Wales, but the questionnaires used to collect information were slightly different between the two countries. However, in the England-only model (see the Supplementary Materials S2 for sub-analyses results), we did investigate if there was an association with bottled water, information that was not collected in the Welsh data, but no association was identified.

Finally, regional variations exist regarding the follow-up, the frequency, version used and method of questionnaire administration and systems to detect common exposures. There are also regional variations in the rate of referrals of specimens to the reference laboratory, for species determination and subtyping. Hence, the rates of outbreak identification and investigation vary [29] and the true size of the *C. hominis* outbreak may have been greater than we detected, as will the exceedances of *Cryptosporidium* spp.

## Conclusions

This work demonstrates the importance of robust surveillance of cryptosporidiosis for timely detection and investigation of possible outbreaks, and the value of using cases of other gastrointestinal infections as controls. Our work identified a number of exposures, including international travel and use of swimming pools, for cases of *C. hominis* in England and Wales compared with cases of *Campylobacter* spp. occurring at the same time period. This study contributes to understanding the 2023 increase in the incidence of *C. hominis* cases, with the causation likely multifactorial, but it does not fully explain the reasons behind it. Further work is needed on the impact of COVID-19, travel and swimming pool exposures, and how weather patterns may affect the burden from cryptosporidiosis, which could be explored through multi-country collaborations. We recommend the development of a standardised questionnaire with clear documentation, training and appropriate resources to ensure consistent follow-up of cases and to enhance questionnaire completion. This will enable rapid investigation of outbreaks and improved understanding of disease dynamics, informing public health actions and improving our existing surveillance.

## Disclaimer

RV is affiliated with the NIHR Protection Research Unit (HPRU) in Gastrointestinal Infections at the University of Liverpool in partnership with the UK Health Security Agency (UKHSA), in collaboration with the University of Warwick, and with the NIHR HPRU in Emerging and Zoonotic Infections at University of Liverpool in partnership with the UKHSA, in collaboration with University of Oxford. The views expressed are those of the authors and not necessarily those of the NIHR, the Department of Health and Social Care, or the UKHSA.

## Supplementary Data

256000 Supplement
