# Supplementary material for: Retrospective case–case study investigation of a significant increase in Cryptosporidium spp. in England and Wales, August to September 2023
Source: Euro Surveill. 2025 Mar 6;30(9):2400493. doi: 10.2807/1560-7917.ES.2025.30.9.2400493 (PMC11887033; doi:10.2807/1560-7917.ES.2025.30.9.2400493)
Supplement: Supplement [file 24-00493_WILLIAMS_Supplement.pdf]

Supplementary materials

This supplementary material is hosted by Eurosurveillance as supporting information alongside the article "Retrospective case-case study investigation of a significant increase in Cryptosporidium spp. in England and Wales, August to September 2023", on behalf of the authors, who remain responsible for the accuracy and appropriateness of the content. The same standards for ethics, copyright, attributions and permissions as for the article apply. Supplements are not edited by Eurosurveillance and the journal is not responsible for the maintenance of any links or email addresses provided therein.

S1 Supplementary methods

Interactions

Interactions were explored between country of travel and the variable for swimming pool use if these variables had a p-value <0.05 in the multivariable model.

Model convergence

To assist with model convergence in the primary analysis and sub-analyses, nAGQ was set to 10 and the "bobyqa" optimizer was used in the generalised linear mixed-effects model. If there were problems with model convergence, then we explored using other optimizers and increasing the value of nAGQ. The final model and all sub-analyses used the "bobyqa" optimizer. Whilst increasing nAGQ was explored, setting it to 0 allowed the sub-analyses to converge for foreign travel and early time period (pre week 37), and so results of these models are less precise.

Sub-analyses

Sub-analyses were conducted for English cases only, those with foreign travel, no foreign travel, children (aged 15 and under), adults (16 years and older) and by time period that cases were reported, so pre week 37 or from week 37 onwards. In the sub-analyses for children and adults, age was included as a continuous variable.

Sensitivity analyses

Age is included as a categorical variable in the primary analysis and all sub-analyses, apart from in the child and adult sub-analyses. Sensitivity analyses were conducted with age included as a continuous variable for the England and Wales and the England-only models. For the child and adult sub-analyses, we also conducted analyses with age squared included in the model. We used AIC to compare the fit of these models.

S2 Supplementary results

Age and sex distribution of cases and comparator cases

Figure 1: Age and sex distribution for cases (n=203)

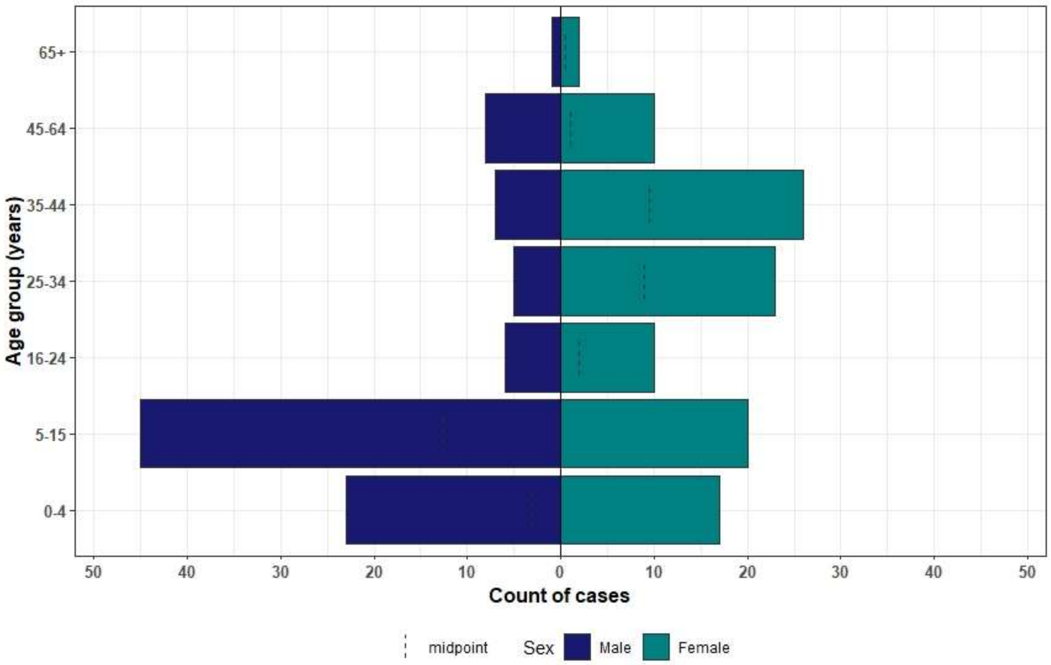

Figure 2. Age and sex distribution for comparator cases (n=612).

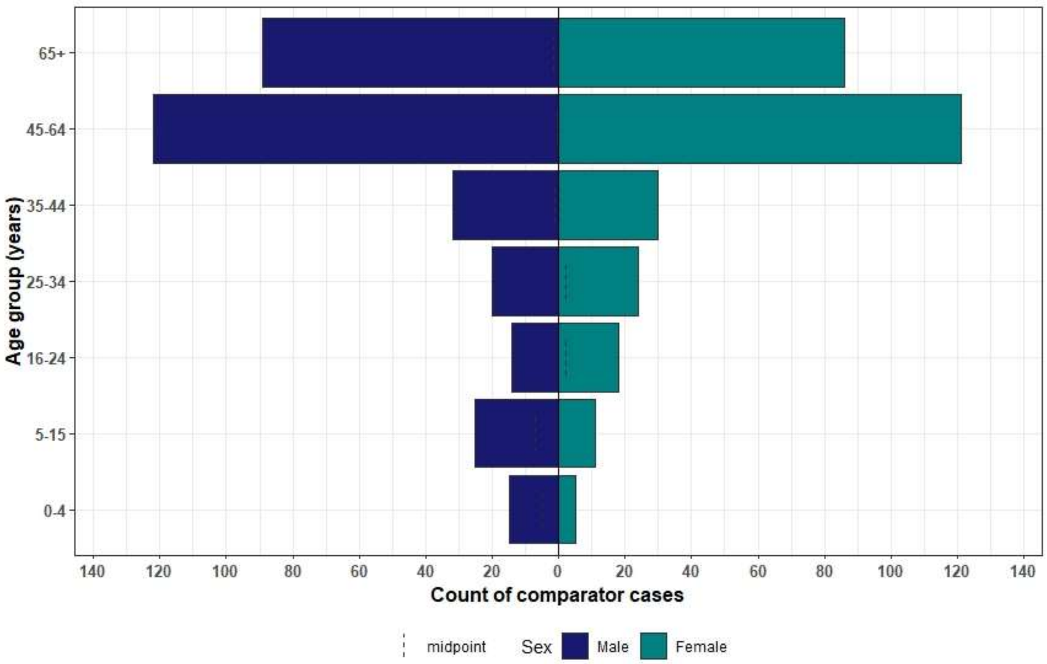

Sub-analyses

England only model

For the England-only multivariable model, the same variables remained in the final model as in the England and Wales model.

Table 1: Final multivariable model for exposures associated with being a case of C. hominis, for England, 14/08/2023 to 30/09/2023.

**Variable**	**N**	**Comparator cases**, N = 459[1]	**Cases**, N = 152[1]	**OR**[2]	**95% CI**[2]	**p-value**
**Age group**	611					
25-34		33 (7.2%)	17 (11%)	—	—	
0-4		18 (3.9%)	31 (20%)	4.09	1.43, 11.7	0.009
5-15		26 (5.7%)	51 (34%)	4.01	1.51, 10.7	0.005
16-24		20 (4.4%)	12 (7.9%)	1.01	0.31, 3.27	>0.9
35-44		46 (10%)	25 (16%)	1.21	0.47, 3.14	0.7
45-64		176 (38%)	13 (8.6%)	0.12	0.05, 0.33	<0.001
65+		140 (31%)	3 (2.0%)	0.07	0.02, 0.27	<0.001
**sex**	611					
Male		243 (53%)	74 (49%)	—	—	
Female		216 (47%)	78 (51%)	1.93	1.08, 3.47	0.027
**Swam in pool**	611	30 (6.5%)	56 (37%)	4.67	2.23, 9.79	<0.001
**Swam in river**	611	4 (0.9%)	5 (3.3%)	6.47	1.10, 38.3	0.039
**Travel - Türkiye**	611	10 (2.2%)	16 (11%)	5.39	1.61, 18.0	0.006
**Travel - Spain**	611	31 (6.8%)	58 (38%)	8.08	3.93, 16.6	<0.001
**Travel - France**	611	5 (1.1%)	5 (3.3%)	9.08	1.74, 47.4	0.009

[1]n (%)

[2]OR = Odds Ratio, CI = Confidence Interval

Foreign travel

For the foreign travel multivariable model, the variables for swimming pool use, travel to Spain and travel to France remained in the model, other than age and sex which are included a priori.

Table 2: Final multivariable model for exposures associated with being a case of C. hominis that had foreign travel, 14/08/2023 to 30/09/2023.

Variable	N	Comparator cases, N = 134[1]	Cases, N = 132[1]	OR[2]	95% CI[2]	p-value
**Age group**	266					
25-34		11 (8.2%)	19 (14%)	—	—	
0-4		2 (1.5%)	19 (14%)	4.94	0.82, 29.9	0.082
5-15		9 (6.7%)	49 (37%)	3.24	0.97, 10.8	0.055
16-24		14 (10%)	12 (9.1%)	0.50	0.14, 1.85	0.3
35-44		11 (8.2%)	22 (17%)	0.75	0.22, 2.54	0.6
45-64		65 (49%)	11 (8.3%)	0.06	0.02, 0.20	<0.001
65+		22 (16%)	0 (0%)	0.00	0.00, Inf	>0.9
**Sex**	266					
Male		65 (49%)	59 (45%)	—	—	
Female		69 (51%)	73 (55%)	1.65	0.77, 3.55	0.2
**Swam in pool**	266	24 (18%)	72 (55%)	5.37	2.20, 13.1	<0.001
**Travel - Spain**	266	38 (28%)	69 (52%)	3.30	1.51, 7.18	0.003
**Travel - France**	266	7 (5.2%)	12 (9.1%)	12.3	2.71, 56.3	0.001

[1]n (%)

[2]OR = Odds Ratio, CI = Confidence Interval

No foreign travel

For the no foreign travel multivariable model, the variables for swimming pool and other water-based activities remained in the model.

Table 3: Final multivariable model for exposures associated with being a case of C. hominis that had no foreign travel, 14/08/2023 to 30/09/2023.

Variable	N	Comparator cases, N = 478[1]	Cases, N = 71[1]	OR[2]	95% CI[2]	p-value
**Age group**	549					
25-34		33 (6.9%)	9 (13%)	—	—	
0-4		18 (3.8%)	21 (30%)	4.19	1.38, 12.8	0.012
5-15		27 (5.6%)	16 (23%)	1.72	0.56, 5.31	0.3
16-24		18 (3.8%)	4 (5.6%)	0.86	0.21, 3.45	0.8
35-44		51 (11%)	11 (15%)	0.71	0.24, 2.08	0.5

Variable	N	Comparator cases, N = 478[1]	Cases, N = 71[1]	OR[2]	95% CI[2]	p-value
45-64		178 (37%)	7 (9.9%)	0.14	0.05, 0.45	<0.001
65+		153 (32%)	3 (4.2%)	0.07	0.02, 0.29	<0.001
**Sex**	549					
Male		252 (53%)	36 (51%)	—	—	
Female		226 (47%)	35 (49%)	1.90	0.98, 3.66	0.056
**Other water-based activity**	549	24 (5.0%)	13 (18%)	2.60	1.04, 6.52	0.041
**Swam in pool**	549	33 (6.9%)	21 (30%)	4.34	1.89, 9.96	<0.001

[1]n (%)

[2]OR = Odds Ratio, CI = Confidence Interval

Children (15 years or younger)

For the children-only model, so for those aged 0-15 years, the only variables remaining in the model were swimming pool use and travel to Spain.

Table 4: Final multivariable model for exposures associated with being a case of C. hominis for children (15 years or younger), 14/08/2023 to 30/09/2023.

Variable	N	Comparator cases, N = 56[1]	Cases, N = 105[1]	OR[2]	95% CI[2]	p-value
**Age**	161	8.0 (2.0, 11.0)	6.0 (3.0, 9.0)	0.95	0.87, 1.04	0.2
**Sex**	161					
Male		40 (71%)	68 (65%)	—	—	
Female		16 (29%)	37 (35%)	1.91	0.77, 4.73	0.2
**Swam in pool**	161	9 (16%)	51 (49%)	5.52	2.14, 14.2	<0.001
**Travel - Spain**	161	2 (3.6%)	37 (35%)	14.7	3.05, 70.8	<0.001

[1]Median (IQR); n (%)

[2]OR = Odds Ratio, CI = Confidence Interval

Adults (16 years and older)

Table 5: Final multivariable model for exposures associated with being a case of C. hominis for adults (16 years and older), 14/08/2023 to 30/09/2023.

Variable	N	Comparator cases, N = 556[1]	Cases, N = 98[1]	OR[2]	95% CI[2]	p-value
**Age**	654	58 (45, 67)	36 (30, 42)	0.93	0.91, 0.95	<0.001
**Sex**	654					
Male		277 (50%)	27 (28%)	—	—	
Female		279 (50%)	71 (72%)	2.14	1.20, 3.83	0.010
**Swam in pool**	654	48 (8.6%)	42 (43%)	6.91	3.50, 13.6	<0.001
**Travel - Spain**	654	36 (6.5%)	32 (33%)	3.82	1.89, 7.72	<0.001
**Travel - France**	654	7 (1.3%)	8 (8.2%)	11.1	2.78, 44.2	<0.001

[1]Median (IQR); n (%)

[2]OR = Odds Ratio, CI = Confidence Interval

Time period – pre-ISO week 37

Table 6: Final multivariable model for exposures associated with being a case of C. hominis for pre-ISO week 37.

Variable	N	Comparator cases, N = 308[1]	Cases, N = 97[1]	OR[2]	95% CI[2]	p-value
**Age group**	405					
25-34		25 (8.1%)	16 (16%)	—	—	
0-4		7 (2.3%)	16 (16%)	3.55	0.89, 14.2	0.074
5-15		17 (5.5%)	31 (32%)	1.81	0.58, 5.66	0.3
16-24		18 (5.8%)	9 (9.3%)	1.12	0.32, 3.86	0.9
35-44		31 (10%)	16 (16%)	0.60	0.21, 1.76	0.4
45-64		127 (41%)	9 (9.3%)	0.10	0.03, 0.29	<0.001
65+		83 (27%)	0 (0%)	0.00	0.00, Inf	>0.9
**Sex**	405					
Male		159 (52%)	44 (45%)	—	—	
Female		149 (48%)	53 (55%)	1.83	0.89, 3.76	0.10
**Swam in pool**	405	28 (9.1%)	48 (49%)	6.09	2.59, 14.3	<0.001
**Travel - Spain**	405	19 (6.2%)	43 (44%)	5.02	2.11, 12.0	<0.001
**Travel - France**	405	6 (1.9%)	6 (6.2%)	6.42	1.16, 35.6	0.033

Variable	N	**Comparator cases**, N = 308[1]	**Cases**, N = 97[1]	OR[2]	95% CI[2]	p-value

[1]n (%)

[2]OR = Odds Ratio, CI = Confidence Interval

Time period – from ISO week 37 onwards

Table 7: Final multivariable model for exposures associated with being a case of C. hominis for ISO week 37 onwards.

Variable	N	**Comparator cases**, N = 304[1]	**Cases**, N = 106[1]	OR[2]	95% CI[2]	p-value
**Age group**	410					
25-34		19 (6.3%)	12 (11%)	—	—	
0-4		13 (4.3%)	24 (23%)	4.03	1.22, 13.4	0.023
5-15		19 (6.3%)	34 (32%)	2.96	0.95, 9.27	0.062
16-24		14 (4.6%)	7 (6.6%)	0.38	0.08, 1.79	0.2
35-44		31 (10%)	17 (16%)	0.79	0.25, 2.51	0.7
45-64		116 (38%)	9 (8.5%)	0.06	0.02, 0.22	<0.001
65+		92 (30%)	3 (2.8%)	0.06	0.01, 0.29	<0.001
**Sex**	410					
Male		158 (52%)	51 (48%)	—	—	
Female		146 (48%)	55 (52%)	2.04	1.03, 4.05	0.040
**Swam in pool**	410	29 (9.5%)	45 (42%)	5.45	2.48, 12.0	<0.001
**Travel - Türkiye**	410	7 (2.3%)	13 (12%)	10.1	2.44, 41.9	0.001
**Travel - Spain**	410	19 (6.3%)	26 (25%)	8.09	3.14, 20.8	<0.001
**Travel - France**	410	1 (0.3%)	6 (5.7%)	92.1	6.55, 1,296	<0.001

[1]n (%)

[2]OR = Odds Ratio, CI = Confidence Interval

Sensitivity analyses

For the primary model, England and Wales, a sensitivity analysis was conducted where age was included as a continuous variable rather than as a categorical variable (table 6) and both models contained the same variables after stepwise analysis. Comparing the model fit for the primary model of England and Wales, the LRT was 0.07 and the AIC for age as a categorical variable was 526 and as

a continuous variable was 526.16. Hence, we have kept age as a categorical variable in the main analyses.

Table 7: Multivariable model for England and Wales with age included as a continuous variable, 14/08/2023 to 30/09/2023.

**Variable**	**N**	**Comparator cases**, N = 612[1]	**Cases**, N = 203[1]	**OR**[2]	**95% CI**[2]	**p-value**
**Age**	815	56 (37, 66)	13 (6, 35)	0.94	0.92, 0.95	<0.001
**Sex**	815					
Male		317 (52%)	95 (47%)	—	—	
Female		295 (48%)	108 (53%)	2.25	1.38, 3.66	0.001
**Swam in pool**	815	57 (9.3%)	93 (46%)	5.47	3.13, 9.55	<0.001
**Swam in river**	815	4 (0.7%)	6 (3.0%)	5.80	1.04, 32.3	0.045
**Travel - Türkiye**	815	11 (1.8%)	17 (8.4%)	3.44	1.18, 10.0	0.024
**Travel - Spain**	815	38 (6.2%)	69 (34%)	5.86	3.19, 10.8	<0.001
**Travel - France**	815	7 (1.1%)	12 (5.9%)	9.91	2.72, 36.0	<0.001

[1]Median (IQR); n (%)

[2]OR = Odds Ratio, CI = Confidence Interval

For the sub-analyses for children, those aged 0-15 years, and for adults, 16 years and older, sensitivity analyses were conducted with age included as a squared variable. After applying the stepwise approach, both of these models contained the same variables as in their respective main analyses with age as continuous variable. When comparing fit of the two models for children, the AICs were similar (age squared 161.01 vs age 161.67). For the adult models, the AIC was smaller for the model with age included as a squared variable (squared 369.38 vs age 374.68).

Sensitivity analyses with age included as age squared variable

Children (0-15 years)

Table 8: Final multivariable model for exposures associated with being a case of C. hominis for children (15 years or younger) with age included as a squared variable, 14/08/2023 to 30/09/2023.

**Variable**	**N**	**Comparator cases**, N = 56[1]	**Cases**, N = 105[1]	**OR**[2]	**95% CI**[2]	**p-value**
**Age squared**	161	64 (4, 121)	36 (9, 81)	1.00	0.99, 1.00	0.2
**Sex**	161					
Male		40 (71%)	68 (65%)	—	—	
Female		16 (29%)	37 (35%)	1.94	0.78, 4.80	0.2

**Variable**	**N**	**Comparator cases**, N = 56[1]	**Cases**, N = 105[1]	**OR**[2]	**95% CI**[2]	**p-value**
**Swam in pool**	161	9 (16%)	51 (49%)	5.45	2.11, 14.1	<0.001
**Travel - Spain**	161	2 (3.6%)	37 (35%)	14.4	2.98, 69.8	<0.001

[1]Median (IQR); n (%)

[2]OR = Odds Ratio, CI = Confidence Interval

Adults (16 years or older)

Table 9: Final multivariable model for exposures associated with being a case of C. hominis for adults (16 years or older) with age included as a squared variable, 14/08/2023 to 30/09/2023.

**Variable**	**N**	**Comparator cases**, N = 556[1]	**Cases**, N = 98[1]	**OR**[2]	**95% CI**[2]	**p-value**
**Age squared**	654	3,307 (2,025, 4,489)	1,296 (900, 1,764)	1.00	1.00, 1.00	<0.001
**Sex**	654					
Male		277 (50%)	27 (28%)	—	—	
Female		279 (50%)	71 (72%)	2.06	1.16, 3.67	0.014
**Swam in pool**	654	48 (8.6%)	42 (43%)	6.29	3.22, 12.3	<0.001
**Travel - Spain**	654	36 (6.5%)	32 (33%)	3.57	1.77, 7.18	<0.001
**Travel - France**	654	7 (1.3%)	8 (8.2%)	11.6	2.85, 47.2	<0.001

[1]Median (IQR); n (%)

[2]OR = Odds Ratio, CI = Confidence Interval
